# Recessive *GNE* Mutations in Korean Nonaka Distal Myopathy Patients with or without Peripheral Neuropathy

**DOI:** 10.3390/genes15040485

**Published:** 2024-04-11

**Authors:** Nasrin Tamanna, Byung Kwon Pi, Ah Jin Lee, Sumaira Kanwal, Byung-Ok Choi, Ki Wha Chung

**Affiliations:** 1 Department of Biological Sciences, Kongju National University, Gongju 32588, Republic of Korea; emutamanna88@gmail.com (N.T.); pi0979@naver.com (B.K.P.); jhmom1010@naver.com (A.J.L.); 2 Department of Biosciences, COMSATS University Islamabad, Sahiwal 45550, Pakistan; sumaira.kanwal@cuisahiwal.edu.pk; 3 Department of Neurology, Samsung Medical Center, Sungkyunkwan University School of Medicine, Seoul 06351, Republic of Korea; 4 Cell & Gene Therapy Institute, Samsung Medical Center, Seoul 06351, Republic of Korea; 5 Samsung Advanced Institute for Health Sciences & Technology, Seoul 06351, Republic of Korea

**Keywords:** biallelic mutations, *GNE*, Korean, Nonaka distal myopathy, whole-exome sequencing

## Abstract

Autosomal recessive Nonaka distal myopathy is a rare autosomal recessive genetic disease characterized by progressive degeneration of the distal muscles, causing muscle weakness and decreased grip strength. It is primarily associated with mutations in the *GNE* gene, which encodes a key enzyme of sialic acid biosynthesis (UDP-N-acetylglucosamine 2-epimerase/N-acetylmannosamine kinase). This study was performed to find *GNE* mutations in six independent distal myopathy patients with or without peripheral neuropathy using whole-exome sequencing (WES). In silico pathogenic prediction and simulation of 3D structural changes were performed for the mutant GNE proteins. As a result, we identified five pathogenic or likely pathogenic missense variants: c.86T>C (p.Met29Thr), c.527A>T (p.Asp176Val), c.782T>C (p.Met261Thr), c.1714G>C (p.Val572Leu), and c.1771G>A (p.Ala591Thr). Five affected individuals showed compound heterozygous mutations, while only one patient revealed a homozygous mutation. Two patients revealed unreported combinations of combined heterozygous mutations. We observed some specific clinical features, such as complex phenotypes of distal myopathy with distal hereditary peripheral neuropathy, an earlier onset of weakness in legs than that of hands, and clinical heterogeneity between two patients with the same set of compound heterozygous mutations. Our findings on these genetic causes expand the clinical spectrum associated with the *GNE* mutations and can help prepare therapeutic strategies.

## 1. Introduction

Nonaka distal myopathy, also known as GNE myopathy with or without rimmed vacuoles, is an uncommon autosomal recessive genetic disorder characterized by progressive muscle weakness and atrophy, in which distal muscles of the limbs are predominantly atrophied. The overall prevalence of Nonaka distal myopathy is estimated to be 1 to 9 per million. Its onset usually occurs during early adulthood, typically in the second and third decades, and presents symptoms such as difficulty walking and foot drop [1,2]. As the disorder progresses, weakness in the upper and lower extremities starts to manifest while the quadriceps, to a certain extent, are spared, leading to impaired mobility resulting in a need for wheelchair assistance [3]. Muscle biopsies of affected individuals have shown protein aggregates, increased acid phosphatase reactivity, congophilic inclusions, rimmed vacuoles, and enhanced lysosomal activity [4,5,6]. Nonaka distal myopathy has been reported in variable populations, including Japanese, Germans, Irish, South Asian, and British populations.

The history of Nonaka distal myopathy dates back to the early 1980s, when Nonaka first reported some cases of Japanese families suffering from distal muscle weakness [7]. Over the years, the genetic locus responsible for causing such symptoms has been mapped to chromosome 9p13.3 [8,9]. A mutation in the *GNE* gene located in the mapped region was first reported in affected individuals of Middle Eastern descent with hereditary inclusion body myopathy [10], followed by Japanese and Italian families [11,12]. To date, more than 200 *GNE* mutations have been reported to be linked to distal myopathies in countries worldwide, with genetic heterogeneity observed across different ethnic groups [1,3,13,14]. Several studies have also reported *GNE* mutations in Koreans as the underlying cause of distal myopathy [15,16,17]. An early study revealed that *GNE* mutations were most frequent in genetically diagnosed Korean patients with recessive myopathy, either in compound heterozygous or homozygous states [15].

The uridine diphosphate-N-acetylglucosamine (UDP-GlcNAc) 2 epimerase/N-acetylmannosamine (ManNAc) kinase (GNE), which is encoded by the *GNE* gene, is a key enzyme in the biosynthesis of sialic acid in vertebrates. The epimerase and kinase domains play important roles in converting UDP-N-acetylglucosamine to ManNAc and phosphorylating ManNAc to ManNAc 6-phosphate, respectively [18,19]. Disruptions in sialic acid production lead to abnormalities in glycoproteins and glycolipids, impacting various cellular processes [2,20]. The resulting aberrations may contribute to cellular dysfunction and muscle degeneration. The formation of rimmed vacuoles in muscle fibers, a distinctive pathological feature, indicates the accumulation of abnormal cellular components [1]. A *GNE* transgenic mouse expressing human *GNE* mutation developed similar features of distal myopathy with rimmed vacuoles [21,22]. Nonaka distal myopathy seems to develop from complicated interactions between genetic mutations and disruptions in cellular structures and processes, ultimately leading to progressive muscle degeneration of this debilitating condition observed in affected individuals. Despite these advances, the exact disease-causing mechanism remains unexplored.

Peripheral neuropathies, such as distal hereditary motor neuropathy (dHMN) and axonal Charcot–Marie–Tooth disease type 2 (CMT2), have been recently reported as an additional clinical feature of the *GNE* mutations [23,24,25,26]. Previtali et al. identified *GNE* mutations in dHMN patients [23], and Zhang et al. identified *GNE* mutations in patients who showed evidence of peripheral nerve involvement and inflammatory cell infiltration [25]. These findings concern a spectrum of clinical features linked to *GNE* mutations, wherein peripheral nerve involvement can lead to progressive distal muscle weakness and disease progression.

Research and genetic studies have provided valuable insights into the mechanisms underlying Nonaka distal myopathy. The significance of studying *GNE* mutations is to improve diagnosis, uncover novel therapeutic targets, and explore potential strategies to correct or compensate for the underlying genetic abnormalities. This study was performed to find *GNE* mutations in distal myopathy patients with or without peripheral neuropathy using whole-exome sequencing (WES). From this genetic study, we identified six unrelated patients with homozygous or compound heterozygous mutations in *GNE*.

## 2. Materials and Methods

### 2.1. Subjects

**Figure 1:**
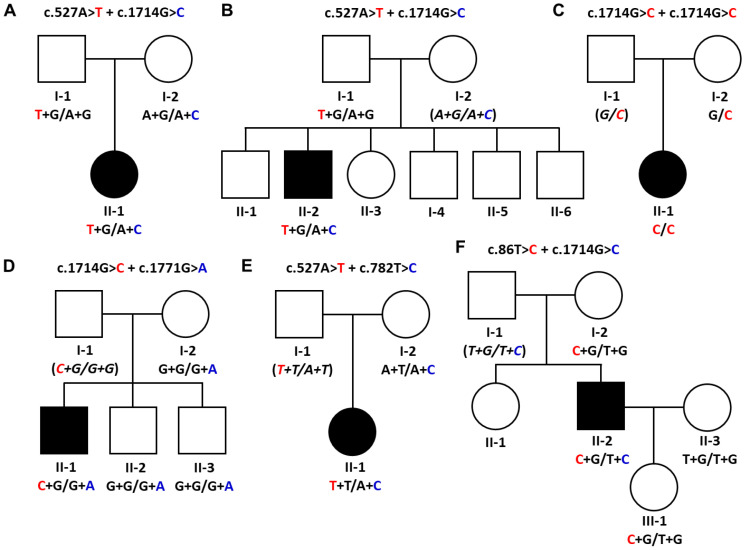
Pedigrees and causative *GNE* mutations in the distal myopathy families. Male and female individuals are represented by squares and circles, respectively. Unfilled symbols indicate unaffected individuals; black-filled symbols indicate affected individuals. Haplotypes of compound heterozygous mutations are provided at the bottom of all the examined individuals. Italic letters in the parentheses are presumptive haplotypes. Red and blue nucleotides indicates rare variant alleles. (**A**) Myo-6 family with c.527A>T (p.Asp176Val) and c.1714G>C (p.Val572Leu) compound heterozygous mutations, (**B**) Myo-8 family with c.527A>T (p.Asp176Val) and c.1714G>C (p.Val572Leu) compound heterozygous mutations, (**C**) Myo-12 family with a homozygous mutation of c.1714G>C (p.Val572Leu), (**D**) Myo-24 family with c.1714G>C (p.Val572Leu) and c.1771G>A (p.Ala591Thr) compound heterozygous mutations, (**E**) Myo-35 family with c.527A>T (p.p.Asp176Val) and c.782T>C (p.Met261Thr) compound heterozygous mutations, and (**F**) Myo-41 family with c.86T>C (p.Met29Thr) and c.1714G>C (p.Val572Leu) compound heterozygous mutations.

This study analyzed six Korean families with distal myopathy with or without peripheral neuropathic symptoms (Figure 1). Among these families, genetic testing was performed in 17 participants, of which 6 of them were affected. All the participants provided written informed consent. This study was conducted by procuring blood samples from the participants admitted at the Samsung Medical Center, Republic of Korea. The institute review boards of Sungkyunkwan University, Samsung Medical Center, and Kongju National University oversaw the study design and authorized this study (SMC_2018-05-102-002 and KNU_IRB_2018-06).

### 2.2. Clinical and Electrophysiological Examinations

To collect clinical information, age at onset, disease duration, and degree of disease disability were investigated in the examined families. Clinical information was measured in the usual standard manner, and tests were performed for motor and sensory disturbances, deep tendon reflexes, and muscle atrophy. The strength of the flexor and extensor muscles was measured using the Medical Research Council (MRC) standard scale. Age at onset was determined by asking patients when symptoms, including distal muscle weakness, first appeared. Sensory disturbances were measured by the degree and severity of pain, temperature, vibration, and posture. For electrophysiological analysis, a peripheral nerve conduction study (NCS) and electromyography (EMG) were performed. The NCS used surface stimulation to measure motor and sensory conduction velocities of the median, ulnar, radial, peroneal, tibial, and sural nerves.

### 2.3. Lower Extremity MRI

The Myo-41 patient underwent lower extremity magnetic resonance imaging (MRI) of the hip, thigh, and lower leg muscles twice at ages 39 and 41. MRI was performed using a 3.0-T MRI system (Skyra, Siemens Healthcare, Frankfurt, Germany). Imaging was performed in the axial field of view (FOV; 524–532 cm, slice thickness 510 mm, slice spacing 50.5–51.0 mm) and coronal FOV (538–540 cm, slice thickness 54–55 mm, slice spacing 50.5–51.0 mm). The following protocol was used in patients: T1-weighted spin echo (SE) (repetition time ([TR]) 5570–5650 ms, echo time ([TE]) 514–520 ms, 512 matrix), T2—weighted SE (TR52, 800–800 ms, 4000 ms, TE596–599 ms, 512 matrix) and fat-suppressed T2-weighted SE (TR53,090–54,900 ms, TE585–599 ms, 512 matrix).

### 2.4. Nucleic Acid Extraction and Exome Sequencing

Genomic DNA (gDNA) was extracted from peripheral blood samples with the QIAmp DNA Blood Midi-kit (Biofact, Daejeon, Republic of Korea). The gDNA was subjected to whole-exome sequencing (WES) using the method of Choi et al. [27]. Sureselect Human All exome 50M kit was utilized for efficient exome capturing (Agilent Technologies, Santa Clara, CA, USA), and the Hiseq 2500 Genome Analyzer (Illumina, San Diego, CA, USA) was used for exome sequencing. Capturing and sequencing of the exome were performed by Macrogen Inc. (Seoul, Korea). The UCSC assembly hg19/GRCh37 and the Genome Data Viewer (https://www.ncbi.nlm.nih.gov/genome/gdv/browser/genome/ accessed on 3 February 2024) were used for the reference sequence and mapping. The GATK and SAMtools were used to identify small nucleotide variants (SNVs). Databases such as the dbSNP, the Genome Aggregation Database (gnomAD; https://gnomAD.broadinstitute.org/ accessed on 3 February 2024), the International Genome Sample Resource (IGSR; https://www.internationalgenome.org/ accessed on 3 February 2024), and the Korean Reference Genome Database (KRGDB) were used to filter rare or novel alleles by keeping the frequency < 0.01. The pathogenicity of SNVs was classified into five grades (pathogenic, likely pathogenic, uncertain significance, likely benign, and benign) based on the guidelines of the American College of Medical Genetics and Genomics and the Association for Molecular Pathology (ACMG/AMP; http://wintervar.wglab.org/ accessed on 1 April 2024). Candidate variants that may be associated with the pathological manifestation of distal myopathy were validated by Sanger sequencing using the SeqStudio Genetic Analyzer (Life Technologies-Thermo Fisher Scientific, Carlsbad, CA, USA).

### 2.5. Conservation and Prediction of 3D Structural Changes of GNE Variants

In silico pathogenicity of the *GNE* variants was predicted using the PolyPhen-2, Mutation Taster, MUpro, and Rare Exome Variant Ensemble Learner (REVEL) programs. Conservation of mutational sites among various organisms was analyzed by Mega-X (ver. 11.0.11; http://www.megasoftware.net/ accessed on 10 January 2024). The GERP++ software (http://genome.ucsc.edu/cgi-bin/hg/ accessed on 10 January 2024) was exploited for evolutionary rate profiling. Predicted 3D structural changes of the wild type and mutant proteins were obtained from the AlphaFold Protein Structure Database (http://alphafold.ebi.ac.uk/ accessed on 17 January 2024), and the obtained 3D structures were then analyzed by the PyMOL Molecular Graphics System version 2.5.5 (http://pymol.org/ accessed on 17 January 2024) [28,29]. To predict the impact of the missense variants on the protein structure, stability, and function, we used a wide range of bioinformatic tools: Missense3D (http://missense3d.bc.ic.ac.uk/ accessed on 17 January 2024), PremPS (https://lilab.jysw.suda.edu.cn/research/PremPS/ accessed on 17 January 2024), DynaMut2 (http://biosig.unimelb.edu.au/dynamut2/ accessed on 18 January 2024), MAESTROweb (https://biwww.che.sbg.ac.at/maestro/web/ accessed on 10 January 2024), and DeepDDG (http://protein.org.cn/ddg.html/ accessed on 10 January 2024).

## 3. Results

### 3.1. GNE Mutations as the Underlying Causes of Myopathy

This study identified five pathogenic or likely pathogenic *GNE* variants in six unrelated patients from the WES data (Figure 1, Table 1). All allele frequency databases, including IGSR, gnomAD, and KRGDB, indicated very low allele frequencies for the selected variants. Three and two variants were evaluated as pathogenic and likely pathogenic by the ACMG/AMP criteria, respectively (Table S1). Five affected individuals showed compound heterozygous mutations, while only one patient revealed a homozygous mutation. For this reason, haplotypes were provided for each mutation’s genotype in all the screened family members (Figure 1). Sanger sequencing was used to screen extended family members for the chosen *GNE* mutations (Figure S1).

Both patients in the Myo-6 and Myo-8 families showed compound heterozygous mutations of c.527A>T (p.Asp176Val) and c.1714G>C (p.Val572Leu). In the Myo-6 family, the affected woman (II-1) carried one mutation (p.Asp176Val) inherited from the father (I-1) and another mutation (p.Val572Leu) from the mother (I-2). The unaffected father and mother were alternatively heterozygous carriers for each of the two mutations, respectively (Figure 1A). In the Myo-8 family, the affected man (II-2) showed both mutations, while his father (I-1) carried only the p.Asp176Val mutation. Thus, it is posited that the unaffected mother (I-2) might have a heterozygous p.Val572Leu mutation (Figure 1B). This combination of heterozygous mutations has been reported twice in Korean patients with distal myopathy [30,31].

An affected woman (II-1) in the Myo-12 family showed a homozygous c.1714G>C (p.Val572Leu) mutation, while the unaffected mother (I-2) had the identical mutation in a heterozygous state (Figure 1C). It is suggested that the unaffected father (I-1) might have the same mutation as a heterozygous state. Although the patient showed a rare homozygous mutation in Koreans, no history of consanguinity could be found in that family. This homozygous mutation has been reported several times in Asian myopathy patients [16,32,33,34].

A set of compound heterozygous mutations, c.1714G>C (p.Val572Leu) and c.1771G>A (p.Ala591Thr), was found in an affected man of the Myo-24 family (Figure 1D). An unaffected mother (I-2) and two younger brothers (II-2 and II-3) revealed only one mutation of p.Ala591Thr. It was suggested that the unaffected father (I-1) might have the p.Val572Leu mutation. These compound mutations have been reported several times [16,35].

In the Myo-35 family, an affected woman (II-1) had compound heterozygous mutations for c.527A>T (p.Asp176Val) and c.782T>C (p.Met261Thr), while the unaffected mother (I-2) had only the p.Met261Thr mutation (Figure 1E). Thus, it is suggested that the unaffected father (I-1) might carry the p.Asp176Val mutation. This is the first report on affected individuals possessing both mutations. However, each mutation of p.Asp176Val and p.Met261Thr has been reported several times with other combinations of heterozygous mutations [16,34,36,37,38,39,40].

**Table 1:** *GNE* variants identified in patients with distal myopathy.

Family ID	Nucleotide Change ^1^	Amino Acid Change ^1^	Main Phenotype	Mutant Allele Frequency				ACMG/AMP	References
				IGSR	gnomAD	ESP	KRGDB		
Myo-6	c.527A>T c.1714G>C	p.Asp176Val p.Val572Leu	DMRV	0.0004 0.0002	0.000495 0.000016	URUR	0.002038 0.002037	P P	[30,31]
Myo-8	c.527A>T c.1714G>C	p.Asp176Val p.Val572Leu	Distal myopathy	0.0004 0.0002	0.000495 0.000016	URUR	0.002038 0.002037	P P	[30,31]
Myo-12	c.1714G>C c.1714G>C	p.Val572Leu p.Val572Leu	Distal myopathy	0.0002	0.000016	UR	0.002037	P	[16,34]
Myo-24	c.1714G>C c.1771G>A	p.Val572Leu p.Ala591Thr	Distal myopathy	0.0002 UR	0.000016 UR	UR UR	0.002037 UR	P LP	[15,35]
Myo-35	c.527A>T c.782T>C	p.Asp176Val p.Met261Thr	Distal myopathy	0.0004 UR	0.000495 UR	UR UR	0.002038 UR	P LP	This study ^2^
Myo-41	c.86T>C c.1714G>C	p.Met29Thr p.Val572Leu	Distal myopathy and dHMN	UR 0.0002	UR 0.000016	UR UR	UR 0.002037	P LP	This study ^3^

Abbreviations: ACMG/AMP: the American College of Medical Genetics and the Association for Molecular Pathology; dHMN: distal hereditary motor neuropathy; DMRV: distal myopathy with rimmed vacuoles; ESP: Exome Sequencing Project; gnomAD: Genome Aggregation Database; IGSR: International Genome Sample Resource; KRGDB: Korean Reference Genome Database; LP: likely pathogenic; P: pathogenic; UR: unreported. ^1^ Reference nucleotide and amino acid sequences are from NM_005476.7 and NP_005467.1. ^2^ This is the first report of the affected individuals encompassing both compound heterozygous mutations, while c.782T>C and c.527A>T have each been reported several times as the genetic causes with other partners of heterozygous mutations [16,34,36,37,39,40]. ^3^ This report represents the initial findings in a patient carrying both mutations, but the individual mutations c.86T>C [15] and c.1714G>C [5,11,32,39] have been reported in other studies.

In an affected man (II-2) from the Myo-41 family, compound heterozygous mutations of c.86T>C (p.Met29Thr) and c.1714G>C (p.Val572Leu) were observed (Figure 1F). The unaffected mother (I-2) and daughter (III-1) had only the p.Met29Thr mutation, and the wife (II-3) showed no mutation. This is the first report of a patient with both mutations. In contrast, the p.Met29Thr and p.Val572Leu mutations have often been reported as the underlying causes of myopathy with other combinations of heterozygous mutations [5,15,16,32,39].

### 3.2. Conservation Analysis and In Silico Prediction of the Mutation Effects

**Figure 2:**
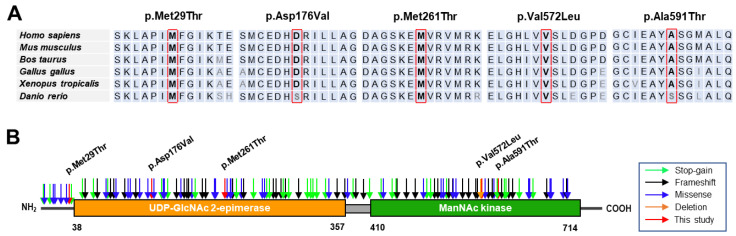
Conservation and location of mutation sites. (**A**) Conservations across different vertebrate organisms. The amino acids at the mutation sites are highlighted in the red boxes. Reference sequences originated from NP_005467.1 (*Homo sapiens*), NP_001177343.1 (*Mus musculus*), NP_001178072.2 (*Bos taurus*), NP_001026603.3 (*Gallus gallus*), NP_001072728.1 (*Xenopus tropicals*), and NP_957177.1 (*Danio rerio*). (**B**) Diagrammatic representation of protein structures and locations of mutations. Identified missense mutations in this study are marked in red arrows, while some reported pathogenic mutations are distinguished using the colors blue (missense), orange (deletion), black (frameshift), and green (stop-gain).

When we predicted the pathogenicity of the identified variants using the MUpro, Mutation Taster, Polyphen-2, and REVEL in silico programs, all the variants were suggested to be pathogenic by the four programs, except for the prediction of p.Asp176Val by PolyPhen-2 (Table 2). The GERP scores were beyond 4.8 at the sites of the variants, which means that the mutation sites are present in highly conserved DNA sequences among mammals. The amino acid sequences in all the mutation sites and their neighboring regions are also well conserved throughout different vertebrates, ranging from mammals to fish (Figure 2A). Three of the identified variants were located in the UDP-GlcNAc 2-epimerase domain (p.Asp176Val and p.Met261Thr) or near the epimerase domain (p.Met29Thr). In contrast, two variants were located in the ManNAc kinase domain (p.Val572Leu and p.Ala591Thr) (Figure 2B). Mutations in the UDP-GlcNAc 2-epimerase domain could show decreased activity of converting UDP-GlcNAc into ManNAc, whereas mutations in the ManNAc kinase domain might be unable to phosphorylate the product processed by the epimerase domain.

**Figure 3:**
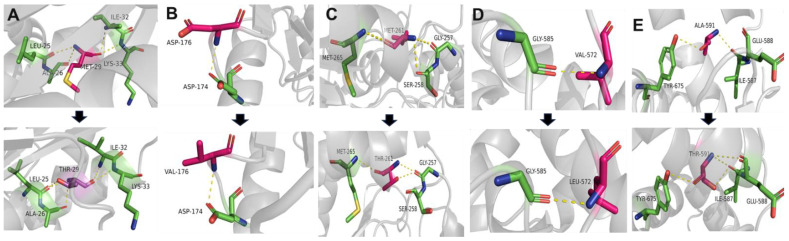
Simulation of structural alterations of the GNE protein due to mutations. Substituted residues are depicted in pink. Carbon atoms in the interacting residues are displayed in green. Hydrogen, nitrogen, oxygen, and sulfur are represented by gray, blue, red, and yellow, respectively. Dashed lines are used to show polar contacts or hydrogen bonds. PyMOL was used to depict the wild-type and mutant residues and find the interacting residues. All contacts were searched for within 4.0 angstroms. The images for the wild and mutated proteins are presented in a top-to-bottom manner. (**A**) p.Met29Thr, (**B**) p.Asp176Val, (**C**) p.Met261Thr, (**D**) p.Val572Leu, and (**E**) p.Ala591Thr.

The structures of the mutated GNE proteins were predicted with the Alphafold structure modeling database, and the predicted 3D structures were visualized and analyzed by the PyMOL software tool (Figure 3). In most cases, conformational changes were observed upon introduction of the mutation. Alteration interactions between substituted residues and surrounding residues were mainly observed in the cases of p.Met261Thr and p.Ala591Thr. An increase in polar interaction was detected in the case of p.Ala591Thr. Structural analysis using Missense3D predicted significant structural damage by p.Val572Leu. This variant was regarded as introducing unfavorably steric clashes. The local clash score of the wild type was 17.37, whereas that of the mutant had a notably elevated score of 36.93. Predicting the effects of the missense variants using MAESTROweb, DynaMut2, PremPS, and DeepDDG showed significant destabilizing effects of the p.Met261Thr, p.Met29Thr, p.Val572Leu, and p.Ala591Thr variants (Table S2).

### 3.3. Clinical Manifestations

The clinical characteristics of the six patients with the *GNE* mutations are shown in Table 3. No family history of distal myopathy was found in any patient. The mean age at onset of leg symptoms was 28.5 ± 8.2 years and that of hand symptoms was 30.8 ± 9.6 years (except for the patient of the Myo-35, who showed no hand symptoms). In the case of the Myo-35 family, four years have passed since lower extremity symptoms appeared, but upper extremity disabilities have not yet appeared. In addition, in most patients, the distal muscles and lower limbs appear to be more severely affected than the proximal muscles and upper limbs, respectively. Decreased or absent deep tendon reflexes were observed in all patients. Foot drop also occurred in all patients. The patient of the Myo-8 family had a very severe disability, including being wheelchair-bound and core muscle involvement, compared to the patient of the Myo-6 family with the same *GNE* mutations of p.Asp176Val and p.Val572Leu. These differences are thought to be partly due to the duration of the disease, which was 3 years in the Myo-6 patient but 10 years in the Myo-8 patient.

All patients were diagnosed with Nonaka distal myopathy based on NCS and EMG imaging. Rimmed vacuoles were observed in the patient of the Myo-6, who was previously reported to have distal myopathy with rimmed vacuoles (DMRV) [30]. The patient of the Myo-41 family showed no compound muscle action potentials (CMAPs) in the peroneal muscles bilaterally and a decrease in CMAPs in the ulnar (7.7 mV, normal range ≥ 8.0 mV), radial (3.8 mV, normal range ≥ 7.0 mV), and tibial nerves (4.5 mV, normal range ≥ 6.0 mV). Additionally, we found decreased motor nerve conduction velocity in the tibial nerve (40.9 m/s, normal range ≥ 41.1 m/s). EMG showed a chronic neuropathy pattern in the distal upper and lower limbs with prolonged duration and increased amplitude of motor unit potentials (MUPs) with vast denervated potentials. Therefore, the patient of the Myo-41 family was diagnosed as having dHMN in addition to distal myopathy. From the filtering analysis of the WES data from the patient of the Myo-41 family, no mutation was selected to be the genetic cause in ~130 peripheral neuropathy-related genes.

### 3.4. Fatty Replacements of Lower Extremity Muscles

**Figure 4:**
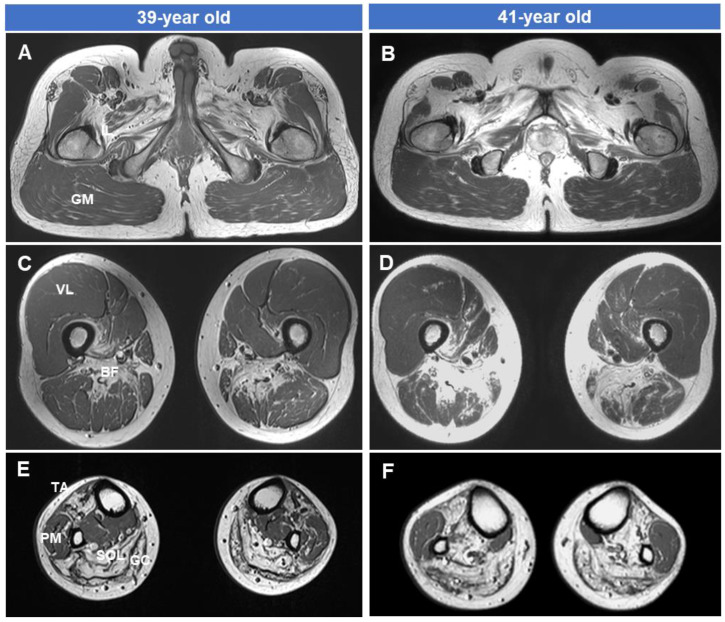
T1-weighted lower extremity MRIs in the male patient of the Myo-41 family. Axial images of the hip (**A**,**B**), thigh (**C**,**D**), and lower leg (**E**,**F**) were taken at 39 (**A**,**C**,**E**) and 41 (**B**,**D**,**F**) years of age. Note the severe fatty involvement of the biceps femoris short head (BF) muscles at the thigh level and the tibialis anterior (TA), soleus (SOL), and gastrocnemius (GC) muscles at the lower leg level. However, the vastus lateralis (VL), gluteus maximus (GM), and peronei muscles (PM) are selectively preserved. This MRI study, conducted over two years, showed rapid progression of muscle degeneration and fat replacement.

The MRI results of the hip (Figure 4A,D), thigh (Figure 4B,E), and lower leg (Figure 4C,F) in the patient of the Myo-41 family at ages 39 and 41, respectively, are shown in Figure 4. There was severe fatty involvement and muscle atrophy of the biceps femoris short head (BF) muscle of the thigh and of the tibialis anterior (TA), soleus (SOL), and gastrocnemius (GC) muscles of the lower leg level. However, selective preservation was observed of the gluteus maximus (GM), vastus lateralis (VL), and peroneal muscles (PM). In the same patient’s T1-weighted image, fatty infiltration and muscle atrophy in the thigh and lower leg levels were more noticeable in the case of a disease duration of 7 years compared to the case of 5 years. Damaged muscles continued to deteriorate, but undamaged muscles remained relatively preserved even after two years. Additionally, an MRI study conducted over two years showed rapid progression of muscle degeneration and fatty replacement.

## 4. Discussion

This study identified *GNE* variants as the underlying causes of autosomal recessive Nonaka distal myopathy in six independent patients. Compound heterozygous mutations were revealed in five patients, and only one patient showed a homozygous mutation. The patients from the Myo-6 and Myo-8 families showed the same compound heterozygous mutations; thus, four different combinations of heterozygous mutations were observed in this study. Compared to some countries, this study showed that the frequency of patients with compound heterozygous mutations was relatively higher (83%) than that of patients with homozygous mutations [39,41,42,43]. This result is consistent with the studies of other recessive genetic diseases in Koreans [44,45]. The identified mutations consisted of five different missense mutations, in which three (p.Met29Thr, p.Asp176Val, and p.Met261Thr) were located within or near the UDP-GlcNAc 2-epimerase domain and two (p.Val572Leu and p.Ala591Thr) were located within the ManNAc kinase domain. The epimerase domain is implicated in the conversion of UDP-N-acetyl glucosamine to ManNAc, and the kinase is responsible for phosphorylating ManNAc to ManNAc 6-phosphate, which is eventually processed into sialylated glycoconjugate [18,19]. All the mutation sites had high GERP scores (>4.8) and were well-conserved among vertebrates. Among the variants, one homozygous mutation and two pairs of heterozygous mutations (p.Asp176Val + p.Val572Leu and p.Val572Leu + p.Ala591Thr) reported as the causes of distal myopathy were evaluated as “pathogenic” [15,16,30,31,32,33,34,35]. However, the other two pairs of heterozygous mutations (c.1714G>C + c.1771G>A and c.527A>T + c.782T>C) were not reported in patients with distal myopathy, although each variant has been reported several times to be pathogenic with combinations of other heterozygous mutations [5,16,32,34,36,37,39,40,46]. These two pairs were determined to be “likely pathogenic”. In silico analysis can reveal the deleterious effects of the missense variants by predictive alteration of the protein’s structure and stability. When the identified mutations were examined for their impact by several in silico programs, they were predicted to be pathogenic. Upon 3D structural modeling of these mutations, the structures of the GNE protein were predictively modified in terms of the polar contacts, such as the hydrogen bonds between neighboring residues and significant destabilizing effects were predicted. These complications might include exaggerated immunological responses, muscle cell degeneration, and disruptions in the signal transduction between cells, which are the classical manifestations of GNE myopathy. However, more studies are needed to ascertain how these mutations impact the function of the enzyme.

In our research participants, p.Val572Leu and p.Asp176Val were found to be the most and second most frequent alleles as the genetic causes (50% and 25%, respectively). These alleles have also been frequently found in previous reports for Korean patients [15,16,47,48]. A Japanese study also reported p.Val572Leu and p.Asp176Val as the first and second frequent pathogenic alleles in patients with Nonaka myopathy [39]. In a cohort of Chinese patients, p.Asp176Val was the most common [40]. However, the common genetic causes of Nonaka myopathy were considerably different by ethnic and regional groups [2,42]. For instance, the most frequent mutations were p.Val727Met in India [42], Met712Thr in Middle Eastern countries [41], and p.Ala662Val in England [49].

The distal muscles and lower limbs showed more severe atrophy than the proximal muscles and upper limbs. We also observed that the onset of weakness in legs (28.5 ± 8.2 years) was slightly earlier than that of the hands (30.8 ± 9.6 years). Moreover, an affected woman (Myo-35), whose leg onset was at 25 years of age, did not develop hand symptoms at that age or at 29 years of age. An affected man in the Myo-8 family showed very severe disability, including being wheelchair-bound and core muscle involvement (aspiration), compared to an affected woman in the Myo-6 family having the same set of compound heterozygous mutations. Respiratory dysfunction is one of the rare, atypical symptoms of Nonaka myopathy [46]. Although this difference in severity was thought to be partly due to the duration of the disease, it suggests the presence of modifying factors that can influence the clinical phenotypes. Phenotypic heterogeneity has frequently been reported in patients with Nonaka myopathy [25,40,43].

For a long time, the unusual peripheral neuropathic symptoms in patients with Nonaka distal myopathy have not received sufficient attention. Recently, several studies have reported peripheral neuropathy in affected persons with *GNE* mutations [23,24,25,26]. Surprisingly, some patients showed a clear association with neuropathy. Therefore, although mutations in the *GNE* gene have been primarily described as being associated with myopathies, the neurological involvement associated with *GNE* mutations may be an underdiagnosed pathology and may influence clinical symptoms and disease progression. In this study, one of the six patients with distal myopathy (Myo-41) was also diagnosed with peripheral neuropathy through neurologic examination and electrophysiological studies. We diagnosed him as having complex phenotypes of dHMN and myopathy.

Lower extremity MRI results taken at 2-year intervals in the affected man (Myo-41) with both symptoms of myopathy and neuropathy showed a pattern of muscle involvement associated with *GNE* mutations. At the thigh level, the biceps femoris short head muscle was damaged earlier and more severely than the vastus lateralis muscle. Additionally, at the lower leg level, fatty replacement occurred earlier and more severely in the anterior compartment muscles (tibialis anterior, extensor halluces, and digitorum longus muscles) and posterior compartment muscles (soleus and gastrocnemius muscles) than in the lateral compartment muscles (peroneus longus and brevis muscles). Follow-up MRI studies over a two-year interval revealed a rapid progression of muscle degeneration and fatty replacement. This finding is consistent with the patient’s clinical manifestations. However, it is interesting to note that the vastus lateralis and peronei muscles, which were not damaged, showed no signs of damage even after 2 years.

Although there are no significant therapeutic drugs approved for Nonaka myopathy, trials for several potential therapeutic tools, including sialic acid supplementation and gene therapy, are currently in progress [6,50]. Long-term safety was assessed for ManNAc, which may provide preliminary evidence for clinical efficacy [51]. In a randomized pilot trial, treatment of 6’-sialyllactose (6SL) significantly increased free sialic acid and improved proximal limb powers, which suggested 6SL as a candidate for Nonaka myopathy therapeutics [52]. Park et al. established multiple isogenic Nonaka myopathy models from human pluripotent stem cells (hPSCs), which showed a mutation-specific drug response [53]. Therefore, understanding the genetic causes will help develop therapeutic strategies for Nonaka myopathy.

## 5. Conclusions

This study identified biallelic *GNE* variants in six patients with Nonaka distal myopathy. Two patients revealed unreported combinations of combined heterozygous mutations. This study also observed some specific clinical features: (a) One patient showed peripheral neuropathy in addition to distal myopathy. (b) The onset of weakness in legs was slightly earlier than that of the hands. (c) Clinical heterogeneity was observed in two patients with the same compound heterozygous mutations. Our findings suggest a loose genotype–phenotype correlation and expand the clinical spectrum associated with *GNE* mutations and will thus help prepare therapeutic strategies.

## Figures and Tables

**Table 2:** Domains and in silico predictions of *GNE* variants identified in this study.

Variant ^1^		Domain	dbSNP	GERP	In silico Prediction ^2^			
Nucleotide	Amino Acid				PP2	MutT	MUp	REVEL
c.86T>C	p.Met29Thr	Epimerase	UR	4.83	0.983 ^*^	1.00 ^*^	−1.00 ^*^	0.93 ^*^
c.527A>T	p.Asp176Val	Epimerase	rs139425890	5.67	0.108	0.99 ^*^	−0.11 ^*^	0.80 ^*^
c.782T>C	p.Met261Thr	Epimerase	UR	5.77	0.996 ^*^	0.94 ^*^	−1.00 ^*^	0.84 ^*^
c.1714G>C	p.Val572Leu	Kinase	rs121908632	5.75	0.968 ^*^	0.96 ^*^	−0.36 ^*^	0.83 ^*^
c.1771G>A	p.Ala591Thr	Kinase	rs752286512	5.75	0.957 ^*^	0.94 ^*^	−0.38 ^*^	0.77 ^*^

Abbreviations: GERP: genomic evolutionary rate profiling score; MUp: MUpro; MutT: Mutation Taster; PP2: PolyPhen-2; REVEL: Rare Exome Variant Ensemble Learner; UR: unreported. ^1^ Reference nucleotide and amino acid sequences are from NM_005476.7 and NP_005467.1, respectively. ^2^ Scores of ~1 (PP2), >0.5 (MutT), <0 (MUp), and >0.5 (REVEL) indicate pathogenic prediction. ^*^ denotes a pathogenic prediction.

**Table 3:** Clinical features of patients with *GNE* mutations.

Family: Patient	Myo-6: II-1	Myo-8: II-2	Myo-12: II-1	Myo-24: II-1	Myo-35: II-1	Myo-41: III-1
Sex	Female	Male	Female	Male	Female	Male
Mutation	p.Asp176Val p.Val572Leu	p.Asp176Val p.Val572Leu	p.Val572Leu p.Val572Leu	p.Val572Leu p.Ala591Thr	p.Asp176Val p.Met261Thr	p.Met29Thr p.Val572Leu
Examined age (yrs)	38	48	25	23	29	38
Onset–leg (yrs)	35	38	20	19	25	34
Onset–hand (yrs)	36	40	22	19	-	37
Family history	No	No	No	No	No	No
Affected muscle	LE/DM → UE	LE/DM → PM/UE	LE/DM&PM → UE	LE&UE/DM	LE/DM&PM	LE/DM → UE
Electromyography	Myopathy	Myopathy	Myopathy	Myopathy	Myopathy	Myopathy, neuropathy
Phenotype	Distal myopathy	Distal myopathy	Distal myopathy	Distal myopathy	Distal myopathy	Distal myopathy, dHMN
Sensory loss	No	No	No	No	No	No
Knee jerk (Right/Left)	+/+	−/−	+/+	+/+	−/−	−/−
Foot drop	Yes	Yes	Yes	Yes	Yes	Yes
Core muscles involved	No	Yes ^1^	No	No	No	No
Wheelchair-bound	No	Yes	No	No	No	No

Abbreviations: dHMN: distal hereditary motor neuropathy; DM: distal muscle; LE: lower extremity; PM: proximal muscle; UE: upper extremity. ^1^ Aspiration sign.

## Data Availability

The data sets generated and analyzed during this study are available from the corresponding author upon reasonable request.

## Supplementary Materials

The following supporting information can be downloaded at: https://www.mdpi.com/article/10.3390/genes15040485/s1, Figure S1: Chromatograms of *GNE* mutations observed in the patients with myopathy; Table S1: Interpretation of the *GNE* gene variants according to the ACMG/AMP criteria; Table S2: Predicted effects of missense mutations using several bioinformatic tools.
