# Supplementary material for: Recessive GNE Mutations in Korean Nonaka Distal Myopathy Patients with or without Peripheral Neuropathy
Source: Genes (Basel). 2024 Apr 11;15(4):485. doi: 10.3390/genes15040485 (PMC11050279; doi:10.3390/genes15040485)
Supplement: Supplementary file 1 [file genes-15-00485-s001.zip › genes-2947643-supplementary.pdf]

Recessive *GNE* Mutations in Korean Nonaka Distal Myopathy Patients with or without Peripheral Neuropathy

Nasrin Tamanna [1], Byung Kwon Pi [1], Ah Jin Lee [1], Sumaira Kanwal [2], Byung-Ok Choi [3,4,5,*] and Ki Wha Chung [1,*]

[1] Department of Biological Sciences, Kongju National University, Gongju 32588, Republic of Korea
[2] Department of Biosciences, COMSATS University Islamabad, Sahiwal, Pakistan
[3] Department of Neurology, Samsung Medical Center, Sungkyunkwan University School of Medicine, Seoul 06351, Republic of Korea
[4] Cell & Gene Therapy Institute, Samsung Medical Center, Seoul 06351, Republic of Korea
[5] Samsung Advanced Institute for Health Sciences & Technology, Seoul 06351, Republic of Korea

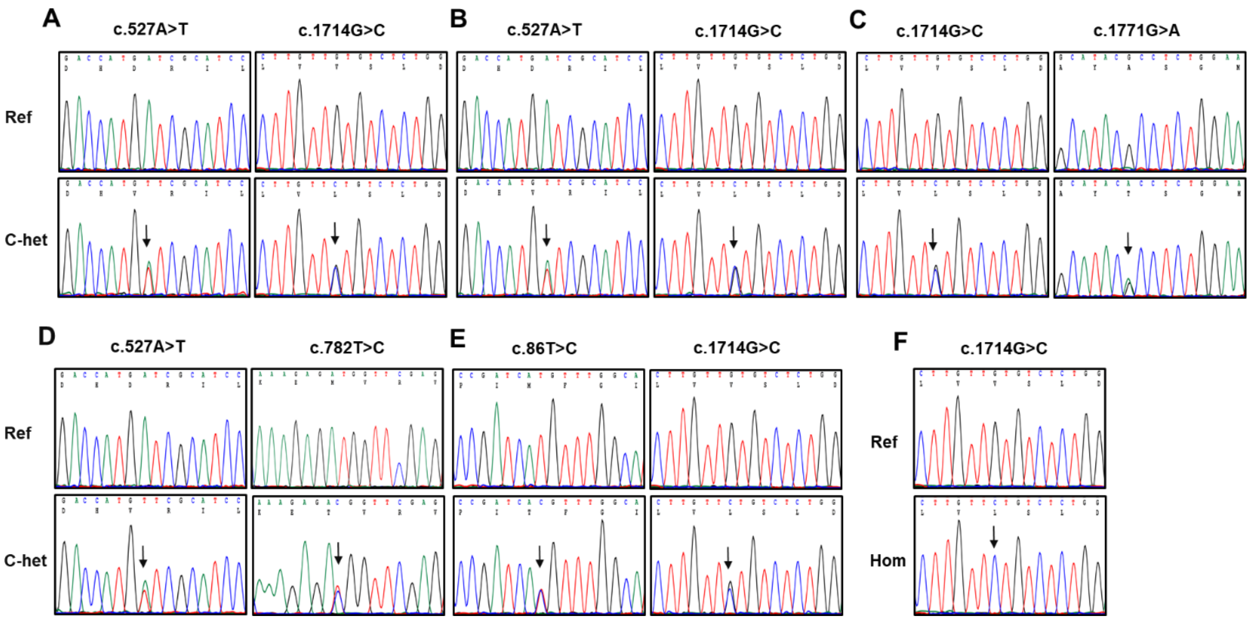

Figure S1. Chromatograms of the *GNE* mutations observed in patients with myopathy. Results were derived via Sanger sequencing, and vertical arrows were employed to pinpoint the mutation sites (Ref: reference allele, C-het: compound heterozygote, Hom: homozygote). (**A**,**B**) c.527A>T and c.1714G>C mutations in families of Myo-6 and Myo-8, (**C**) c.1714G>C and c.1771G>A mutations in pedigree Myo24, (**D**) c.527A>T and c.782T>C mutations in pedigree Myo-35, (**E**) c.86T>C and c.1714G>C mutations in pedigree Myo-41, and (**F**) c.1714G>C homozygous mutation in pedigree Myo-12.

Table S1. Interpretation of the *GNE* gene variants according to the ACMG/AMP criteria.

Variants		Criteria	Evaluation
**Nucleotides**	**Amino Acids**		
c.86T>C	p.Met29Thr	PM1+PM2+PM3+PP1+PP3+PP4	P
c.527A>T	p.Asp176Val	PM1+PM2+PM3+PP1+PP3+PP4+PP5	P
c.782T>C	p.Met261Thr	PM1+PM2+PM3+PP1+PP3+PP4	LP
c.1714G>C	p.Val572Leu	PM1+PM2+PM3+PP1+PP3+PP4+PP5	P
c.1771G>A	p.Ala591Thr	PM1+PM2+PM3+PP1+PP3+PP4	LP

Abbreviations: ACMG-AMP: The American College of Medical Genetics and Genomics and the Association for Molecular Pathology, LP: likely pathogenic, P: pathogenic.

Table S2. Predicted effects of missense mutations using several bioinformatic tools.

Variants	DDG (kcal/mol)[a]			
	**MAESTROweb**	**DynaMut2**	**PremPS**	**DeepDDG**
p.Met29Thr	0.39	-1.28	-2.47	-2.21
p.Asp176Val	-0.15	0.06	-0.23	-0.187
p.Met261Thr	0.33	-1.72	-2.17	-0.909
p.Val572Leu	0.03	-0.34	-0.39	-1.095
p.Ala591Thr	0.22	-1.67	-1	-0.762

Abbreviations: MAESTROweb: Mutation Analysis for the Estimation of Stability Changes in Proteins Web Server; DynaMut2: Dynamic mutation 2; PremPS: Prediction of the Effects of Missense Mutations using Protein Structure; DeepDDG: Deep Learning-based $\Delta\Delta G$ (delta delta G).

[a] DDG or $\Delta\Delta G$ (kcal/mol): difference in Gibbs free energy between two states (wild-type and mutant). A negative value ($\Delta\Delta G < 0$) suggests that the mutant protein destabilizes the protein structure and function compared to the wild type while a positive value ($\Delta\Delta G > 0$) indicates that the mutant stabilizes the protein.
